# The relationship between some endometrial secretion cytokines and in vitro fertilization 

**Published:** 2015-09

**Authors:** Mohammad Ehsan Rahiminejad, Amirhossein Moaddab, Mehrnoosh Ebrahimi, Soghra Rabiee, Alireza Zamani, Mohammad Ezzati, Alireza Abdollah Shamshirsaz

**Affiliations:** 1 *Department of Obstetrics & Gynecology, Hamadan University of Medical Sciences and Health Services, Hamadan, Iran.*; 2 *Department of Obstetrics and Gynecology, Baylor College of Medicine, Houston, Texas, USA.*; 3 *Department of Immunology and Hematology, Hamadan University of Medical Sciences and Health Services, Hamadan, Iran.*; 4 *Department of Obstetrics and Gynecology, UT Southwestern Medical Center, Dallas, TX, USA.*

**Keywords:** *Cytokine*, *In vitro fertilization*, *Outcome*, *Endometrium*, *Implantation*

## Abstract

**Background:**

Endometrial secretion analysis is a non-invasive and promising method in evaluation of endometrial receptivity.

**Objective:**

The aim of the present study was to assess the relationship between the success rate of IVF procedures and some endometrial secretion cytokines, including interleukin-1β (IL-1β), tumor necrosis factor (TNF-α), interferon gamma-induced protein 10 (IP-10), and monocyte chemoattractant protein (MCP).

**Materials and Methods:**

In a prospective cohort study, 50 women selected for IVF met the study inclusion criteria. All the patients underwent endometrial secretion aspiration prior to embryo transfer. The level of IL-1β, TNF-α, IP-10 and MCP were analyzed by enzyme-linked immunosorbent assay method using special standard kits. To detect successful implantation and pregnancy patients underwent serum human chorionic gonadotropin measurements and ultrasound evaluation.

**Results:**

Five samples were excluded. Nine women (20%) had successful clinical pregnancies, which resulted in live birth. Other 36 women (80%) were classified as failed pregnancy. Comparison of cytokine levels showed lower concentrations of TNF-α, IP-10, and MCP in the group with successful clinical pregnancy compared to the group with failed pregnancy (p=0.007, 0.005 and 0.001, respectively). However, no significant difference was revealed in IL-1β levels between two groups (p=0.614).

**Conclusion:**

The current study suggested that lower concentrations of TNF-α, IP-10, and MCP in endometrial secretions might be associated with improved endometrial receptivity and IVF outcome. Regarding IL-1β, no statistically significant differences were seen between the groups with and without successful pregnancy.

## Introduction

Infertility is a common condition, affecting marital relationship, mental health and the couples’ quality of life. Recent advances in assisted reproductive technologies (ARTs) such as In Vitro Fertilization (IVF) provide more efficient methods for infertility treatment (1-3). Despite the increasing prevalence, most infertility treatment modalities are very expensive and impose a heavy financial burden on families. Moreover, a significant number of IVF procedures does not result in a live birth (3-5).

Successful implantation depends on the quality of the embryo and the endometrial receptivity. (6) Cytokines produced by fetal and uterine mucus are responsible for regulating interactions between the mother and fetus, which improve uterus receptivity by controlling the expression of proteins involved in the adhesion process (7, 8). The known cytokines and growth factors that may have contributed to the increased receptivity of human endometrial implantation include leukemia inhibitory factor, interleukin-1, keratinocyte growth factor and colony stimulating factor-1. An ideal biomarker for increased endometrial receptivity must be present in the endometrium, should be close to the site of implantation and should appear during the implantation period and disappear after it (9).

Identification of such biomarkers may provide more information about implantation in both normal and ART cycles. Furthermore, the identification of these markers might be useful in determining the best time for embryo transfer. The aim of the present study was to assess the relationship between the success rate of IVF procedures and some endometrial secretion cytokines, including interleukin-1β (IL-1β), tumor necrosis factor (TNF-α), interferon gamma-induced protein 10 (IP-10), and monocyte chemoattractant protein (MCP). These cytokines were chosen based on controversial results of previous studies focused on describing the immunological dialogue between a vital embryo and a receptive endometrium in implantation (1, 6-8).

## Materials and methods

**Study design**

This prospective non-randomized study were perfomed at Fatemieh Women’s Hospital from September 2011 to March 2013. Fifty women less than 35 years of age who were candidates for IVF due to tubal factor infertility were enrolled after providing sufficient explanation and obtaining informed consent.The study protocol was approved by the Research Ethics Committee of Hamadan University of Medical Sciences. To confine the effects of factors which affect IVF outcome, all women met the study inclusion criteria, including a) normal menstrual cycles between 25 to 35 days, b) female aged less than 35 years old, c) BMI less than 30, d) TSH <10 on the third day of the cycle and the number of antral follicles more than 6 on the third day ultrasound, and e) normal semen analysis. Also, exclusion criteria were as follows: a) metabolic and/or endocrine disorders (diabetes, metabolic syndrome, PCOS and Thyroid Disorders) b) previous gynecological/pelvic surgery except for salpingectomy, c) more than once previous failed IVF cycle, and d) smoking.

**IVF technique**

To stimulate ovulation, recombinant follicular stimulating hormone (rFSH) (Puregon, Organon, Germany) was injected daily from the third day of the menstrual cycle with an appropriate dosage. In order to prevent premature luteinization, daily injection of gonadotropin-releasing hormone (GnRH) antagonist (Ganirelix acetate, Organon, Netherland) (450 IU once daily) was considered from the fifth day of the IVF cycle. In order to stimulate final oocyte maturation, an intramuscular dose (10000 IU) of human chorionic gonadotropin (hCG) (Pregnyle, Organon, Germany) was injected. A maximum of three embryos were transferred, 4-5 days after the ovarian puncture. Embryo transfer was performed in the luteal phase. An embryo with stage-specific cell size, <10% of fragmentation and no multinucleation was considered as grade A. Embryos with stage-specific cell size for the majority of cells, 10–25% of fragmentation and no evidence of multinucleation considered as grade B and embryos with cell size not stage-specific, severe fragmentation (more than 25%) and evidence of multinucleation counted as grade C (10).

During the first 5 days after embryo transfer, three hCG injections (each containing 5000 IU) and intramuscular progesterone (Progestan, Organon, Germany) injection (100 mg/day) were administered for luteal phase support.

Serum β-hCG was measured 10 days after embryo transfer in order to detect implantation. In case of a positive test result, the test was repeated 48 hours later. A double increase in β-hCG level was considered as successful implantation. Women with successful implantation underwent ultrasound evaluation of the pregnancy from four weeks after oocyte retrieval. Pregnancy was defined by visualization of an embryonic sac at vaginal ultrasound examination at 5 weeks after embryo transfer. Losing pregnancy products diagnosed by ultrasound any time after pregnancy detection was considered as a failed IVF cycle.

**Aspiration and analysis of endometrial secretions**

Aspiration of endometrial secretion was performed by the method described by Boomsma (1). Endometrial secretions were aspirated by insertion a trans-cervical catheter and gentle suction with a 2-mL syringe prior to embryo transfer. In previous studies, the safety of the uterine secretions aspirated prior to embryo transfer have shown [11]. All the samples were stored in liquid nitrogen at a temperature of -80^°^C. The level of cytokines including interleukin-1β (IL-1β), tumor necrosis factor (TNF-α), interferon gamma-induced protein 10 (IP-10), and monocyte chemoattractant protein (MCP) in the aspirated fluid were analyzed by enzyme-linked immunosorbent assay (ELISA) method using special standard kits. (R&D Systems, Minneapolis, Minnesota).

**Statistical analysis**

Values were mean±SD/median (25; 75th percentiles) and data were analyzed statistically by χ^2^ test or Mann–Whitney U-test. Statistical Package for the Social Sciences, version 19.0, SPSS Inc, Chicago, Illinois, USA (SPSS software) was used for statistical analysis. P<0.05 was considered statistically significant.

## Results

Fifty patients were recruited for this study. Five samples were excluded because of contact bleeding in the course of sampling. Thus, aspirated endometrial secretions of 45 enrolled women were analyzed to assess levels of IL-1β, TNF-α, IP-10, and MCP. According to serum β-hCG levels and ultrasound studies, 9 women (20%) had successful clinical pregnancies which resulted in live birth. Other 36 women (80%) were classified as the group with failed pregnancy. There was no significant difference between women with successful pregnancy and failed pregnancy in terms of baseline and clinical characteristics (Table I). The concentrations of TNF-α, IP-10, and MCP in the group with successful clinical pregnancy were significantly different compared to the group with failed pregnancy (p-values 0.007, 0.005 and 0.001, respectively) (Table II). However, no significant difference was revealed in IL-1β levels between two groups. 

**Table I:** Baseline and clinical characteristics in groups with successful pregnancy and failed pregnancy

**Characteristics**	**Successful pregnancy (n = 9)**	**Failed pregnancy (n = 36)**	**p-value**
Age, yr (Mean±SD)	28.0 ± 3.3	29.7 ± 3.5	0.13
Duration of infertility, yr (Mean±SD)	5.7 ± 2.2	5.9 ± 2.6	0.69
Number of transferred embryos (Mean±SD)	2.2 ± 0.8	2.2 ± 0.9	1.00
**Grade of transferred embryo**			
A*	9 (100%)	29 (80.6%)	0.06
B∆	0	6 (16.7%)	-
C┼	0	1 (2.7%)	-

* Embryo with stage-specific cell size, <10% fragmentation and no multinucleation

∆ Embryo with stage-specific cell size for the majority of cells, 10–25% fragmentation and no evidence of multinucleation

┼ Embryo with cell size not stage-specific, severe fragmentation (25%) and evidence of multinucleation

**Table II:** Comparison of cytokine levels in aspirated endometrial secretions in with successful pregnancy and failed pregnancy

**Cytokine**	**Successful pregnancy (n = 9)**	**Failed pregnancy (n = 36)**	**p-value**
TNF-α*	13.5(10.3;230.45) ■	25.1(17.9;32.7)	<0.01
IL-1β∆	363.7(102.3;636.4)	49.2(35.3;887.2)	0.61
MCP┼	250.5(134.2;372.5)	1050.3(106.3;1995.6)	<0.01
IP-10○	298.2(267.6;407.4)	634.7(290.7;996.3)	<0.01

* Tumor necrosis factor-α

∆ Interleukin-1β

┼ Monocyte chemoattractant protein

○ Interferon gamma-induced protein 10

■ All levels were measured in pg/mL and represents median (25; 75th percentiles)

## Discussion

The current study suggested that lower concentrations of TNF-α, IP-10, and MCP in endometrial secretions were associated with improved uterine receptivity and IVF outcome. Regarding IL-1β, no statistically significant difference was seen between the groups with and without successful pregnancy.

The interactions between maternal immune system and conceptus tissues at implantation are necessary for successful implantation and progression to pregnancy (12). It seemed that these interactions at the level of endometrium regulated cytokines concentrations and Th1/Th2 ratio. Recent studies have shown that some of the secreted inflammatory cytokines not only make no disturbance in implantation, but also are necessary for endometrial reconstruction and modulate the events subsequent to implantation by recruiting the macrophages and dendritic cells (13-16). Some studies hypothesized that slight damage to the endometrium in women with repeated IVF failure leaded to a greater clinical pregnancy rate by modifying endometrial secreted cytokines (17-20).

Previous studies reported some controversial results on the role of different cytokines in providing a proper physiological function for implantation (1, 6-8). TNF-α is not only a key regulator in interactions prior to the implantation, but also higher levels of it plays a crucial role in some reproductive diseases such as endometrial infections and recurrent spontaneous abortions (21-24). Boomsma *et al* study revealed a positive relationship between successful pregnancy and higher levels of TNF-α in endometrial secretions. Similar to earlier animal studies, we found that TNF-α concentrations in pregnant women with successful pregnancy were significantly lower than those with IVF failure (25).

While previous studies have shown that inappropriate expression of IL-1β was associated with repeated abortions and pregnancy failure, the current study failed to find any statistically significant difference between the study groups (25-28).

In a study by Boomsma et al. MPC1 and IP10 levels had significant negative and positive association with initial implantation of embryos, respectively (25). Some studies showed that the embryo expressed some receptors for MPC1 and IP10 (29-35). Furthermore, it was suggested that MPC1 was a strong absorber and activator of uterine natural killer cells, which were associated with abortion and infertility (32-33). Given the relationship between higher rates of implantation with lower levels of these cytokines, these findings were probably consistent with the results of the current study.

Comparing to other studies, the chance to achieve a successful IVF in this study was low (10, 36). We determined the concentrations of the endometrial secretions by ELISA because of its feasibility and regarding the results of previous studies, which demonstrated the similar results of endometrial cytokine measurements by immunohistochemistry, ELISA and semiquantitative reversed transcription-polymerase chain reaction (semiquantitative RT-PCR) (37). In addition to theoretical uncertainties, some factors such as technical differences between this study and previous ones in secretions aspiration or timing of aspiration as well as lower volume of samples could be possible reasons for this difference.

Considerable evidence suggests that pregnancy is a complex event related to complex immunological processes. While infertility and failed ART cycles are tied to emotional distress, further understanding of this phenomenon might lead to more control on factors contributing to the success and failure of pregnancy process and maximize the estimation of success rate (38). The non-invasive nature of analysis of uterine secretion aspiration enabled the researchers of this study to evaluate the uterine receptivity before the embryo transfer in IVF process without inflicting any damage comparing to alternative methods such as endometrial dating. 

## Conclusion

The current study suggests that lower concentrations of TNF-α, IP-10, and MCP in endometrial secretions might be associated with improved endometrial receptivity and IVF outcome. This non-invasive approach may improve understanding of immunological events, which embryo experiences during its early days.
